# Usefulness of peanut and hazelnut molecular components for the diagnosis of nut allergy. Our experience in clinical practice

**DOI:** 10.1186/2045-7022-1-S1-P100

**Published:** 2011-08-12

**Authors:** Giuseppina Rotiroti, James Gardner, Katrien Coppens, Jennifer Parker

**Affiliations:** 1 Royal Free NHS Trust, London, UK

## Background

Children with moderate/severe Eczema frequently develop sensitisations to several foods leading to specific elimination diets. A positive test to nuts is often considered a potential risk for anaphylaxis. Dietary restriction and a constant fear of potential anaphylaxis has a significant impact on patients of life. Component Resolved diagnosis is not yet widely available in the UK and despite the numerous recent publications in the field the experience in clinical practice remains limited in our population. We have recently introduced this methodology in the evaluation of patients attending our paediatric allergy clinic modifying our diagnostic decision pathway by incorporating the results of these new specific component IgEs. Previously some cases would not have been challenged based on published positive predictive values. We report here our 6 months experience with the use of Ara-h2 and Cor-a8 in the evaluation of children with a positive allergy test to nuts.

Amongst all the children investigated 12 (4 female and 8 males) mean age 10.5 (range 2-14) tested negative for Ara h2 and or Cor a8. On the bases of the negative test results all underwent food challenges with Peanut and or hazelnut despite having a positive SPT and/or specific IgE to these nuts. All of these children passed a supervised graded challenge with up to 15 grams of the nut as per validated protocols.

## Specifically

Eight children had positive allergy test to peanut (mean SPT diameter 4.5mm - range 2-7mm)( Specific IgE to Peanut mean 6.75, range 0.4-23.2). Eight children had positive Specific IgE to Hazelnuts (mean value 15.69 range 0.66-90.2)4 of them had positive SPT. Post challenge all the 12 children were able to reintroduce the nuts uneventfully in their diet.

A mini food allergy quality of life questionnaire was performed pre/post challenge demonstrating a significant improvement in quality of life. Our observation is limited by the very small number of patients involved nevertheless it underlines the usefulness of component resolved diagnosis in the investigation of patients with food allergy.

